# Endocannabinoid 2-arachidonoyl glycerol increases the transcription of *daf-7* in ASI neurons

**DOI:** 10.17912/micropub.biology.000056

**Published:** 2018-11-19

**Authors:** Celina Galles, Gastón M Prez, Diego de Mendoza

**Affiliations:** 1 Laboratorio de Fisiología Microbiana, Instituto de Biología Molecular y Celular de Rosario (IBR), CONICET, Facultad de Ciencias Bioquímicas y Farmacéuticas, Universidad Nacional de Rosario, 2000, Rosario, Argentina

**Figure 1.**
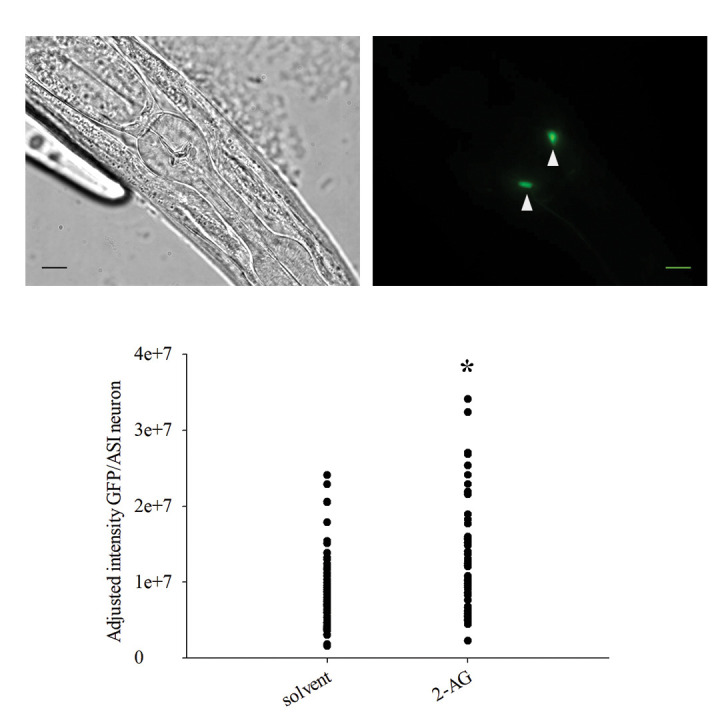


## Corrections

Corrections to this article were made. These corrections were recorded in an Erratum published on Aug 23, 2019.

## Description

*daf-7* expression in ASI neurons is increased when nematodes are grown in the presence of endocannabinoid 2-arachidonoyl glycerol (2-AG). Left upper panel: representative image of reporter strain FK181 *ksIs2 [Pdaf-7::GFP+rol-6(su1006)]* captured under GFP epifluorescence microscopy. Right upper panel: same individual showing bright field microscopy together with GFP signal. Gray/black triangles mark ASI neurons expressing *daf-7*. Scale bars correspond to 0.03 mm. Lower panel: point plot of the adjusted GFP intensity/ASI neuron values gathered from worms treated either with the carrier solvent or with 2-AG. 2-AG treatment significantly increases *daf-7* expression in ASI neurons. (*) indicates statistically significant difference with solvent control condition; the difference in the median values of the relative adjusted GFP fluorescence/ASI neuron between the two groups is greater than would be expected by chance; there is a statistically significant difference between solvent and 2-AG (Mann-Whitney Rank Sum Test, p=<0.001). The number of independent experiments carried out was three. The total number of ASI neurons analyzed was 70 for each condition.

## Methods

Worms were raised on solid NGM plates supplemented with bacteria and the corresponding supplement for each condition tested. *daf-7* expression was monitored following the protocol described by (Myers, 2012). Briefly, synchronized L1 larvae were fed with *Escherichia coli* HT115(DE3) supplemented with either 2-arachidonoyl glycerol (2-AG) 100 µM or with an equal volume of carrier solvent (acetonitrile) for 1,5 h at 20 ºC. GFP fluorescence viewed with confocal microscopy in ASI neurons of L1 FK181 animals. L1 images were captured with Zeiss LSM880 scan head on an axio observer Z1 inverted microscope with a 60x 1.4 AN oil immersion objective. A laser line 488 nm of an argon ion laser was used for the excitation, the detection was done in a GaAsP spectral detector with a

bandwidth between 508 and 588 nm. GFP intensity in ASI neurons was quantified using NIH Image J software. GFP intensity in each ASI cell body was subtracted from the intensity of a similarly sized background selection to get the adjusted GFP intensity /ASI neuron value.

## Reagents

Strain FK181 *ksIs2* *[Pdaf-7::GFP+rol-6(su1006)]*

2-arachidonoyl glycerol (Cayman Chemical)

Acetonitrile (Merck)
