# Erratum: Endocannabinoid 2-arachidonoyl glycerol increases the transcription of *daf-7* in ASI neurons

**DOI:** 10.17912/micropub.biology.000161

**Published:** 2019-08-23

## Description

Galles, C; Prez, GM; de Mendoza, D (2018). Endocannabinoid 2-arachidonoyl glycerol increases the transcription of *daf-7* in ASI neurons. microPublication Biology. 10.17912/micropub.biology.000056 first published online Jul 29, 2018 @ 02:37; corrected after publication Aug 22, 2019.

In the version of the microPublication originally published online, the name of the second author was misspelled. The correct spelling is **Gastón M. Prez**. This mistake has been corrected in the html and PDF versions of the microPublication.

